# The potential of Sijunzi decoction in the fight against gastrointestinal disorders: a review

**DOI:** 10.3389/fphar.2025.1464498

**Published:** 2025-03-04

**Authors:** Liangjun Yang, Zheng Fang, Jiajie Zhu, Xiaofang Li, Bo Yang, Haiyan Liu, Feiyan Lou

**Affiliations:** ^1^ Department of Gastroenterology, Tongde Hospital of Zhejiang Province (Zhejiang Academy of Traditional Chinese Medicine), Hangzhou, China; ^2^ Science and Technology Innovation Center, Guangzhou University of Chinese Medicine, Guangzhou, China; ^3^ Department of Pharmacy, Tongde Hospital of Zhejiang Province (Zhejiang Academy of Traditional Chinese Medicine), Hangzhou, China; ^4^ Endoscopy Center, Tongde Hospital of Zhejiang Province (Zhejiang Academy of Traditional Chinese Medicine), Hangzhou, China

**Keywords:** Sijunzi decoction (SJZD), gastrointestinal disorders, traditional Chinese, functional dyspepsia, irritable bowel syndrome, tumor

## Abstract

Sijunzi Decoction (SJZD) is a traditional Chinese medicine formula widely used in the treatment of gastrointestinal disorders. Clinical studies have substantiated the efficacy of SJZD in managing conditions such as functional dyspepsia, chronic gastritis, gastric cancer, irritable bowel syndrome, colorectal cancer, and ulcerative colitis. Despite its proven effectiveness, the precise mechanisms by which SJZD operates remain incompletely understood. In this study, we undertake a systematic review of both the clinical applications and the mechanistic underpinnings of SJZD in the context of gastrointestinal disease treatment. Research indicates that SJZD functions through a spectrum of mechanisms including the regulation of intestinal flora, alleviation of inflammation, modulation of immune responses, and facilitation of mucosal repair in the treatment of gastrointestinal ailments. This comprehensive analysis aims to provide a clearer understanding of how SJZD benefits patients with gastrointestinal disorders.

## Graphical Abstract

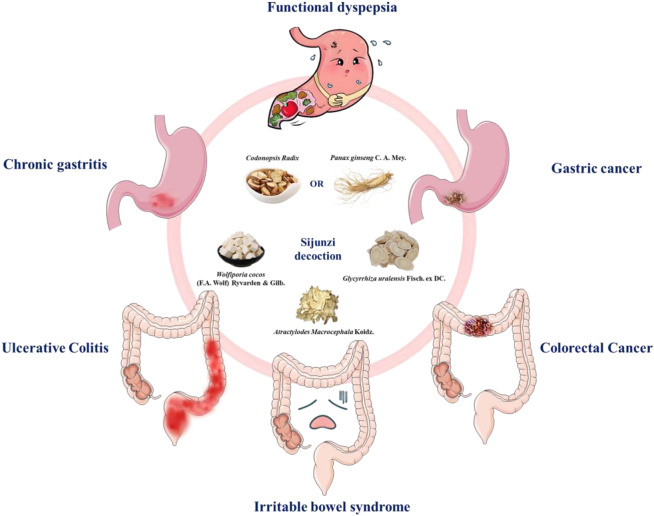

## Introduction

The digestive system, which consists of the gastrointestinal tract, liver, pancreas, and gallbladder, facilitates the digestion of food—a crucial process that converts food into nutrients used by the body for energy, growth, and cellular repair. Gastrointestinal diseases, such as functional gastrointestinal diseases, inflammatory bowel disease, and tumors, significantly impact a large segment of the population, leading to a range of uncomfortable symptoms, increased morbidity, and substantial healthcare costs (Peery et al., 2015). Gastrointestinal diseases are prevalent, with an estimated prevalence rate of 16% for functional dyspepsia (FD) (Ford et al., 2020), over 50% for chronic gastritis (Sipponen and Maaroos, 2015), and 9.2% for irritable bowel syndrome (IBS) (Oka et al., 2020). The inflammatory bowel diseases, primarily including Crohn’s disease and ulcerative colitis (UC), are chronic inflammatory disorders of the gastrointestinal tract. In China, there has been a significant increase in the prevalence and incidence of inflammatory bowel disease over the past 3 decades, which provides challenges for the medical field (Park and Cheon, 2021). Additionally, among gastrointestinal malignancies in China, colorectal and gastric cancers rank second and fifth in terms of incidence, while they rank third and fourth in mortality, respectively (Han et al., 2024). Overall, digestive diseases remain a major contributor to the global healthcare burden, with little to no decline in prevalence or incidence observed in recent years (Wang et al., 2023). Given the substantial impact of these conditions, intervention in patients with digestive diseases is critically important for alleviating symptoms and enhancing their quality of life. However, developing effective drug-based therapies for gastrointestinal diseases presents considerable challenges, particularly due to the unclear etiology of many of these disorders (Halama and Haberkorn, 2020; Mishra et al., 2020; Singh et al., 2022). Addressing this complexity is essential for improving patient outcomes and managing the growing burden of gastrointestinal diseases in the healthcare system.

Traditional Chinese medicine (TCM), developed in China, is an ancient system that extensively examines human physiology, pathology, disease diagnosis, and treatment, with a rich historical background of effectively addressing digestive disorders. Central to this practice is the use of herbal medicine, which is regarded as a vital approach for managing a range of conditions, including functional gastrointestinal diseases (Tan et al., 2020), inflammatory bowel disease (Zhang S. et al., 2022), and malignancies such as gastric and colorectal cancers (Chen J.-F. et al., 2023; Dai Z. et al., 2024). Sijunzi decoction (SJZD), a classical formula in TCM, is composed of four Chinese herbs: *Panax ginseng* C. A. Mey. (or *Codonopsis radix.*), *Wolfiporia cocos* (F.A. Wolf) Ryvarden and Gilb., *Atractylodes macrocephala* Koidz., and *Glycyrrhiza uralensis* Fisch. ex DC. In the SJZD, *P. ginseng* C. A. Mey. (or *C. radix.*) takes the role of the Emperor herb, being the primary ingredient for replenishing Qi and strengthening the spleen and stomach. *Atractylodes macrocephala* Koidz. acts as the Minister herb, supporting the spleen and drying dampness to amplify *C. radix*‘s effects on Qi and spleen reinforcement. *Wolfiporia cocos* (F.A. Wolf) Ryvarden and Gilb. is the Assistant herb, which works in conjunction with *A. macrocephala* Koidz. to further strengthen the spleen and eliminate dampness. Lastly, *G. uralensis* Fisch. ex DC. serves as the Messenger Herb, harmonizing the middle, supplementing Qi, and coordinating the actions of the other herbs within the formula. Together, these four herbs create a balanced and effective composition for tonifying the spleen and stomach.

This formulation has been widely utilized to address spleen deficiency, which presents symptoms such as anorexia, diarrhea, loose stools, and systemic discomforts including fatigue, weakness in the limbs, and cold extremities. A randomized, double-blind, placebo-controlled, multi-center clinical trial has demonstrated that SJZD can alleviate fatigue symptoms and enhance overall health status in patients with chronic fatigue syndrome (Dai L. et al., 2024). SJZD is primarily used to treat gastrointestinal diseases, including chronic gastritis, functional gastrointestinal disorders, UC, and gastrointestinal carcinomas, based on its effects of strengthening the spleen and replenishing Qi in accordance with TCM theory. Over the past 2 decades, an increasing number of clinical studies have reported the application of SJZD in patients with gastrointestinal diseases. Concurrently, a series of experimental studies have been conducted to elucidate the mechanisms by which SJZD treats gastrointestinal diseases. However, the clinical application, pharmacodynamics, and molecular mechanisms of SJZD in the treatment of gastrointestinal diseases have not yet been systematically analyzed, and the common points of SJZD in gastrointestinal diseases have not been revealed. In this article, we review and summarize previous clinical and experimental studies on SJZD for gastrointestinal disorders, as well as insights into its pharmacological effects, aiming to illustrate the advantages and promote the application of TCM in the treatment of gastrointestinal diseases.

## Methodology

In this review, we aim to summarize and evaluate recent evidence primarily drawn from clinical and experimental research, as well as systematic reviews, regarding the efficacy of SJZD in the treatment and management of gastrointestinal disorders. A comprehensive literature search was conducted, focusing mainly on publications from PubMed and ScienceDirect, limited to articles written in English. The keyword “sijunzi” was employed for searches on both databases.

The inclusion criteria encompassed evidence from randomized controlled trials, systematic and narrative reviews, observational studies, nonclinical studies, case reports, and expert opinions published over the past 24 years (2000–2024). In contrast, study protocols, duplicate articles, and conference abstracts were excluded from consideration.

## Sijunzi decoction

As an empirical decoction, Sijunzi decoction was recorded in the Taiping Huimin Hejiju Fang of the Song Dynasty (published around 1110 A.D.). It consists of *P. ginseng* C. A. Mey. (Renshen) or *C. radix* (Dangshen), *W. cocos* (F.A. Wolf) Ryvarden and Gilb. (Fuling), *A. macrocephala* Koidz. (Baizhu), and *G. uralensis* Fisch. ex DC. (Gancao).

*Panax ginseng* C. A. Mey. is one of the most important traditional herbs and healthy foods in East Asian herbal remedies, with a history spanning over 2,000 years. The roots and rhizomes of *P. ginseng* C. A. Mey. are utilized in traditional medicine to treat diseases by replenishing Qi (Zhang et al., 2024). Modern research has identified the main chemical components of ginseng, which primarily include ginsenosides, ginseng polysaccharides, and ginseng polypeptides. Pharmacological studies indicate that ginseng possesses strong anti-inflammatory and immunoregulatory effects in the intestinal system (Zhao et al., 2024). Additionally, a meta-analysis has demonstrated that ginseng consumption is associated with a significantly reduced risk of gastric and colorectal cancer (Jin et al., 2016). This may be due to ginseng’s ability to inhibit the proliferation, growth, invasion, and metastasis of tumor cells, induce tumor cell apoptosis, and ultimately suppress tumor initiation and progression (Ni et al., 2022; Zhao et al., 2022).

*Codonopsis radix* is an herb that has historically been used as a cost-effective substitute for the more expensive *P. ginseng* C. A. Mey. As a widely recognized medicinal plant in the field of TCM, *C. radix* possesses the ability to replenish Qi, strengthen the spleen, and nourish the blood. This herb contains a diverse array of chemical constituents, including flavonoids, polyacetylenes, triterpenes, steroids, alkaloids, resinous substances, and other biologically active compounds. Substantial evidence demonstrates that *C. radix* exhibits diverse biological activities, including immunomodulatory, antitumor, anti-inflammatory, and anti-fatigue properties (Luan et al., 2021). Furthermore, extracts of *C. radix* possess extensive pharmacological properties, such as protecting the gastrointestinal mucosa, exhibiting anti-ulcer effects, demonstrating antitumor activity, regulating endocrine functions, improving hematopoietic activity, and providing cardiovascular protection (Dong et al., 2023).

*Wolfiporia cocos* (F.A. Wolf) Ryvarden and Gilb, commonly known as *Poria cocos*, is a well-known fungus that has been used as a traditional medicine and functional food in China for over 2,000 years. The primary bioactive constituents of *Poria cocos* include polysaccharides, triterpenoids, steroids, fatty acids, and enzymes. Clinically, it is used to treat spleen-deficiency syndrome, often accompanied by symptoms such as digestive disorders, diarrhea, indigestion, and vomiting. Numerous studies have demonstrated that *Poria cocos* offers several beneficial effects, including intestinal protection, modulation of intestinal flora, anti-inflammatory, and antitumor activity (Zou et al., 2021; Wang et al., 2022). As a result, it is considered effective in the treatment of conditions such as IBS (Yang et al., 2023), colitis (Lan et al., 2023), and gastric cancer (Wang et al., 2022).

The rhizomes of *A. macrocephala* Koidz. have traditionally been utilized to invigorate the spleen and replenish qi for the treatment of various diseases. The primary chemical constituents of *A. macrocephala* Koidz. include terpenoids, polysaccharides, alkynes, flavonoids, and steroids. A significant body of research has highlighted the beneficial effects of *A. macrocephala* Koidz. on gastrointestinal diseases. Studies have confirmed that *A. macrocephala* Koidz. and its extracts possess multiple pharmacological activities, including immune enhancement (Xiang et al., 2020), antitumor (Liu et al., 2022), antioxidant (Bailly, 2021), and anti-inflammatory effects (Gu et al., 2019), which have therapeutic potential for gastrointestinal tumors (Chen T. et al., 2023; Choi et al., 2024b), chronic gastritis (Yang S. et al., 2020), UC (Cheng et al., 2023), and IBS (Choi et al., 2024a).

The dried roots and rhizomes of *G. uralensis* Fisch. ex DC., commonly known as Gan-Cao in Chinese, are widely used as herbal medicine worldwide. It is regarded as an “essential herbal medicine” in TCM due to its ability to reduce toxicity and enhance the effectiveness of other medicinal plants when used in combination. The primary chemical constituents of licorice include triterpenoid saponins, flavonoid glycosides, free phenolic compounds, polysaccharides, coumarins, and alkaloids. Currently, it is extensively employed in the treatment of respiratory, liver, and gastrointestinal diseases, owing to its hepatoprotective, anti-inflammatory, antiviral, and antioxidant properties (Jiang et al., 2020; Li et al., 2020).

In summary, the herbs in SJZD exhibit a wide range of pharmacological activities, including immunomodulation, antitumor effects, anti-inflammatory properties, and gastrointestinal protection, thereby addressing a spectrum of gastrointestinal disorders and contributing to the overall health.

## Sijunzi decoction in upper gastrointestinal disorders

### Functional dyspepsia

FD, located in the gastroduodenal region, is among the most common functional gastrointestinal disorders. The prevalence of FD has been steadily increasing, significantly impacting patients’ quality of life. The main contributing factors to FD include abnormal gastrointestinal dynamics, heightened visceral sensitivity, *Helicobacter pylori* infection, disturbances in intestinal flora, and psychological influences. This disorder is characterized by a diverse range of clinical manifestations, such as early satiety, postprandial fullness, and epigastric pain or burning, which seriously affect patients’ quality of life and impose a heavy social and economic burden. In modern medicine, the treatment options for FD predominantly involve gastrointestinal motility agents, therapies aimed at alleviating visceral hypersensitivity, and medications targeting anxiety and depression. However, the efficacy of these treatments remains unsatisfactory (Lacy et al., 2023). TCM is a promising alternative, offering a holistic approach that may provide specific advantages in addressing the complex nature of FD. Based on the symptomatology of functional dyspepsia in TCM, it is commonly described as “distension and fullness,” “stomach pain,” and “retention” (Speciality Committee of Digestive DiseasesChinese Association of Integrative Medicine, 2011). Meta-analysis has shown that TCM formulas tend to yield better outcomes in alleviating overall dyspeptic symptoms (Chu et al., 2018). According to TCM, SJZD has been utilized in accordance with Chinese medicine for managing symptoms of indigestion. A meta-analysis has shown that SJZD-based prescriptions could be effective in treating FD, with no significant adverse effects detected (Wang Y. et al., 2021). However, due to the potential for bias, further validation of the benefits of SJZD for FD treatment necessitates the implementation of standardized, large-scale, and rigorously designed RCTs. These findings suggest that SJZD-based therapies hold promising potential for treating FD.

### Chronic gastritis

Chronic gastritis is a chronic inflammatory response of the gastric mucosa that can be attributed to various factors such as *H. pylori* infection, medication usage, stress, and autoimmune processes. Generally, it presents as bloating, epigastric pain, indigestion, appetite loss, and other symptoms, considerably compromising patients’ quality of life and increasing their susceptibility to developing cancer (Quan et al., 2018). Despite its prevalence, therapeutic approaches for chronic gastritis primarily focus on symptomatic treatment, as no specific Western medicine has been identified, particularly for chronic atrophic gastritis (Yan-Rui et al., 2024). Studies have confirmed that TCM is effective in alleviating the clinical symptoms of patients with chronic gastritis and can also impede the progression of chronic atrophic gastritis towards gastric cancer (Qin et al., 2013; Yang L. et al., 2020). As a classic TCM decoction, a clinical study has proved that SJZD can significantly improve the scores of the histopathology of chronic gastritis and *H. pylori* clearance rate (Gan et al., 2017). Additionally, several scholars have employed a modified version of SJZD for the treatment of chronic atrophic gastritis, and their findings indicate the efficacy of modified SJZD in alleviating fatigue and tiredness symptoms among chronic atrophic gastritis patients (Tian et al., 2019). Furthermore, a meta-analysis has shown that SJZD is an effective treatment option for chronic atrophic gastritis, as it improves clinical outcomes, enhances quality of life, and increases the eradication rate of *H. pylori,* as well as levels of GAS-17, PGI, and PGR in patients with chronic atrophic gastritis (Huang and Shao, 2024).

High gastric acid secretion is correlated with the manifestation of discomforting symptoms in individuals with chronic gastritis. *In vitro* experiments have substantiated the antacid properties of SJZD (Wu et al., 2010), suggesting a potential link to alleviating the distressing symptoms of this condition. Additionally, through network pharmacology analysis, it has been predicted that SJZD may alleviate chronic gastritis by suppressing the inflammatory response of peripheral blood leukocytes. Subsequent experimental studies have further validated the efficacy of SJZD in ameliorating both local gastric inflammation and inflammation in peripheral blood leukocytes (Wang et al., 2020). Gastric precancerous lesions are considered to be crucial steps in the progression from chronic atrophic gastritis to gastric cancer. Early identification, management, and surveillance of gastric precancerous lesions are imperative for preventing gastric cancer. According to proteomics and metabolomics analyses, research has demonstrated that SJZD possesses the capacity to hinder the progression of gastric precancerous lesions by regulating oxidative phosphorylation (Zhu et al., 2024). Additionally, modified formulas derived from SJZD, such as Weipiling decoction (Yang et al., 2022) and Weiwei decoction (Hong et al., 2024), have exhibited specific therapeutic effects in managing precancerous gastric lesions. These results demonstrated that SJZD is effective in the treatment of chronic gastritis.

### Gastric cancer

Gastric cancer, a leading cause of cancer-related deaths globally, is ranked as the fifth most prevalent malignant tumor worldwide (Thrift et al., 2023). Despite advancements in stomach cancer treatment in recent years, the mortality rate associated with this disease remains significant. Hence, finding novel therapeutic approaches for gastric cancer holds exceptional significance. TCM is widely used in gastric cancer patients, especially in Asia. A retrospective cohort study conducted in Taiwan from 1997 to 2010 indicates that Chinese herbal medicine enhances the overall survival of gastric cancer patients (Hung et al., 2017). These findings establish a foundation for future research exploring the effectiveness of TCM as a therapeutic approach for gastric cancer.

SJZD has been widely utilized as a therapeutic approach for the long-term treatment of gastric cancer. A retrospective analysis has demonstrated that combining enteral nutrition with SJZD can effectively treat precachexia in cancer patients through the alleviation of inflammatory response, improvement of nutritional status, and enhancement of performance (Li et al., 2021). Additionally, a meta-analysis was conducted to evaluate the efficacy and safety of SJZD combined with enteral nutrition in GC patients, resulting in significant improvements in albumin, prealbumin, transferrin, immunoglobulin, CD3^+^, CD4^+^, and CD4^+^/CD8^+^ (Chen et al., 2020). Furthermore, treatment with SJZD inhibited cell growth and triggered apoptosis in gastric cancer cells. This effect was achieved by suppressing the expression of gastric cancer stem cell markers, as well as by reducing the nuclear accumulation and DNA binding activity of β-catenin (Jia et al., 2018; Li Y.-J. et al., 2022). CMTM2, an immune-related gene within the CMTM family, plays a significant role in tumor progression, and prognosis in gastric cancer (Qian et al., 2020). *In vitro* investigations reveal that SJZD markedly attenuates the proliferation, migration, invasion, and cancer stem cell-like characteristics of gastric cancer cells through the upregulation of CMTM2 expression (Li X. et al., 2022). Network pharmacology analysis predicted that SJZD may exert anti-gastric cancer effects via a comprehensive mechanism involving multiple compounds, targets, and pathways. Subsequent experimental findings have supported these predictions, demonstrating that SJZD can effectively suppress tumor growth and induce apoptosis in gastric tumor cells. These effects are mediated by the downregulation of key genes such as VEGFA, iNOS, COX-2, and Bax/Bcl2, as well as the inhibition of p-PI3K and p-AKT expression levels (Ding et al., 2022). Overall, the integrated approach of network pharmacology and experimental validation highlights the potential of SJZD as a therapeutic option for gastric cancer treatment.

## Sijunzi decoction in lower gastrointestinal disorders

### Irritable bowel syndrome

IBS is a chronic functional gastrointestinal disorder that presents with abdominal pain related to defecation or alterations in bowel habits. IBS is characterized by various gastrointestinal symptoms, including bloating, abdominal pain, urgency, and diarrhea or constipation, along with altered bowel habits, without any organic abnormalities. The conventional management of IBS typically includes lifestyle modifications, dietary interventions, and pharmacological treatments. Pharmacological treatment options include the regulation of gastrointestinal motility, supplementation with probiotics and prebiotics, as well as the use of antidepressants. However, the effectiveness of these treatments is frequently found to be unsatisfactory (Huang et al., 2023). TCM has shown effectiveness in treating IBS, addressing clinical symptoms such as abdominal pain, distension, and bowel habits, while also improving quality of life. As a promising approach, TCM has the potential to target dysmotility, visceral hypersensitivity, and the gut-brain axis by increasing NPY levels and reducing serotonin, making it a viable option for treating IBS (Xiao et al., 2015; Liu et al., 2023).

SJZD has been utilized in the treatment of IBS, specifically IBS with diarrhea (IBS-D). A meta-analysis revealed that the combined use of modified SJZD and Tongxie Yaofang significantly enhanced the overall clinical effectiveness rate for IBS-D when compared to conventional Western medicine treatments (Yan et al., 2023). The gut microbiota, functioning as a distinct organ with well-defined roles, plays a pivotal role in the development and severity of IBS. Alterations in the microbiota composition are significant factors contributing to the persistence of IBS symptoms (Canakis et al., 2020). Experimental evidence has demonstrated that a purified homogeneous polysaccharide obtained from SJZD exhibits immunomodulatory effects by modulating the abundances of nine genera of intestinal bacteria (Gao et al., 2018). To investigate the specific interactions between SJZD and the intestinal microbiota-derived from patients with IBS-D, an *in vivo* cocultivation system was created. The findings demonstrated that SJZD effectively restored the dysbiosis of the intestinal microbiota and improved the disrupted neurotransmitter metabolism associated with the key symptoms of IBS-D (Xia et al., 2022). In summary, SJZD exhibits therapeutic potential for treating IBS-D by modulating gut microbiota, thereby underscoring its efficacy in comparison to traditional treatments.

### Colorectal cancer

Colorectal cancer ranks as the third most prevalent cancer and the second leading cause of cancer-related deaths worldwide. While the incidence and mortality rates of colorectal cancer have decreased in recent decades, epidemiological studies indicate a potential increase in its occurrence among individuals under the age of 50 (Thanikachalam and Khan, 2019). Considering that the symptoms of the disease typically only manifest in the advanced stage, the implementation of active screening and treatment represents an effective approach to diagnose and treat the condition. The primary treatments for colorectal cancer currently include surgery, radiotherapy, chemotherapy, immunotherapy, and targeted therapy. However, challenges such as surgical complications, chemotherapy resistance, toxic side effects, and elevated rates of metastasis and recurrence significantly compromise patients’ quality of life (Pilkington et al., 2022). Consequently, research into more effective treatment options has become a focal point in this field. Chinese medicine has been utilized in the treatment of colorectal cancer. A cohort study conducted in China revealed that Chinese herbal medicines might mitigate the risk of recurrence and metastasis in patients with stage II and III colorectal cancer (Tang et al., 2022). This emerging evidence highlights an important frontier in the fight against colorectal cancer, suggesting that integrative treatment strategies incorporating TCM could significantly enhance patient prognosis and improve quality of life.SJZD has been used as a classical decoction in the treatment of digestive malignant tumor (Wu and Xuan, 2007). Through network pharmacology analysis, researchers have discovered that SJZD exerts its therapeutic effects on colorectal cancer by modulating multiple targets and pathways. A study specifically investigated the core genes involved in the therapeutic mechanism of SJZD for colorectal cancer, revealing that SJZD influences protein binding in colon cancer by altering the expression of HSPB1, IGFBP-3, and SPP1 (Du et al., 2022). Furthermore, *in vitro* and *in vivo* experiments have confirmed that SJZD can induce apoptosis and autophagy in colorectal cancer cells through the PI3K/Akt/mTOR pathway (Shang et al., 2023). To identify potential therapeutic targets of SJZD, microarray analysis was conducted on patients with colorectal cancer who were undergoing treatment with SJZD. The results indicated that KLF4, which showed a significant correlation with reduced overall survival and recurrence rates, may serve as a potential therapeutic target of SJZD for the treatment of colorectal cancer (Jie et al., 2017). NK cells play a crucial role in cancer immunosurveillance by targeting and killing tumor cells, holding great potential for the therapy of gastrointestinal cancers (Wang F. et al., 2021). A study has shown that SJZD enhances the expression levels of death receptor 4 and death receptor 5 through modulation of P53 expression. This increase in receptor levels consequently enhances the sensitivity of colon cancer cells to NK cell-mediated killing, resulting in the inhibition of colon cancer growth (Wang et al., 2024). The liver is a frequent site of metastasis for colon cancer. A study has proved that modified SJZD demonstrates the ability to inhibit liver metastasis in colon cancer by activating the innate immune system (Zhou et al., 2019). These findings offer a potential complementary and alternative therapy for colon cancer.

### Ulcerative colitis

UC is a multifaceted, chronic immune-mediated inflammatory bowel disease that typically initiates in the rectum and can progress proximally to affect the entire colon (Feuerstein et al., 2019). Common symptoms exhibited by UC patients include frequent bowel movements, presence of mucus and pus, blood in stool, diarrhea, abdominal pain and discomfort, tenesmus, as well as weight loss. Conventional treatments for UC—such as corticosteroids, amino-salicylates, immunomodulatory agents, and biological therapies—are widely utilized in clinical practice. However, these medications have significant limitations, particularly due to their undesirable side effects (Kucharzik et al., 2020). In TCM, UC is classified under the “dysentery” category based on its symptomatology. Clinical research has shown that TCM granules are superior to placebo in inducing clinical remission and promoting mucosal healing among patients with moderately active and 5-ASA-refractory UC (Shen et al., 2021). Recent studies investigating the mechanisms by which TCM treats UC have been steadily increasing. These studies indicate that TCM can effectively inhibit the onset and progression of UC through its anti-inflammatory and antioxidant properties, while also modulating the body’s immune response and other related factors (Zheng et al., 2022).

As a classical Chinese herbal formula, SJZD has been extensively utilized and clinically proven effective in the treatment of UC. Research findings have revealed that SJZD restores the microbial homeostasis and intestinal barrier integrity in UC by inhibiting the abundance of the phylum Proteobacteria and the genus Escherichia-Shigella, alleviating colon tissue damage, and enhancing the expression of tight junction proteins (Wu et al., 2023). The mucosal epithelium plays a crucial role in maintaining the balance of the intestinal ecosystem. Experimental studies have shown that SJZD exhibits a protective effect on the intestinal barrier against TNBS-induced colitis in rats and TNBS-damaged Caco-2 cells *in vitro* (Lu et al., 2017). As an essential substrate for nucleotide synthesis in epithelial cells and a major energy source, l-glutamine is widely used in the treatment of gastrointestinal diseases to alleviate mucositis. Research has demonstrated that the combination of l-glutamine with SJZD is more effective than using l-glutamine alone in ameliorating the severity of diarrhea, histopathological damage, and the disruption of villus and crypt structures in the intestinal mucosa following continuous 5-Fu injections in mice (Qu et al., 2020). As an immune-mediated inflammatory disorder affecting the colon, immune dysregulation plays a crucial role in the pathogenesis of UC. To investigate the impact of SJZD on the immune system in UC, a research study was conducted. The findings demonstrated that SJZD effectively ameliorated the DSS-induced inflammatory response in UC rats by increasing the content of secretory IgA in the intestinal mucosa and elevating the levels of IL-2 in the intestinal tissue (Yu et al., 2016). In summary, SJZD primarily exerts its effect in the treatment of UC by promoting mucosal repair and regulating mucosal immune function.

## Pharmacological mechanisms of SJZD for the treatment of gastrointestinal disorders

**FIGURE 1:**
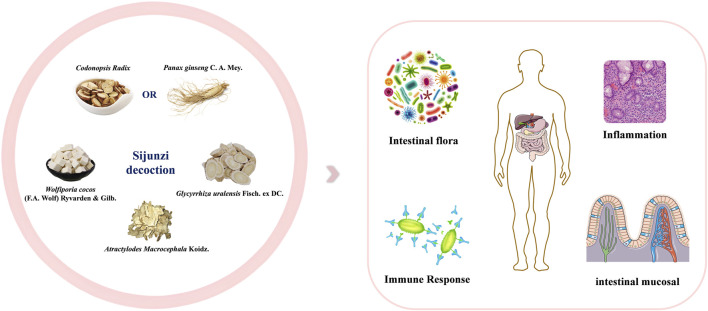
The mechanism of action of SJZD in the treatment of gastrointestinal disorders.

Over the past few decades, a variety of *in vivo* and *in vitro* experimental studies have enhanced our understanding of the mechanisms underlying the effectiveness of SJZD in treating gastrointestinal disorders. This section offers a summary of the different mechanisms of action, which encompass regulating intestinal flora, reducing inflammation, modulating the immune response, and promoting mucosal repair (Figure 1). Table 1 shows detailed information of the pharmacological studies carried out on SJZD.

**TABLE 1:** Mechanism of action of SJZD in treatment of gastrointestinal disorders.

Disease	*Codonopsis radix* or *radix ginseng*	Mechanism	Apply styles	References
None	*Codonopsis radix*	increase the abundance of *Bifidobacterium_pseudolongum*	*in vivo*	Zhang et al. (2022b)
Dysbacteriotic diarrhea	*Codonopsis radix*	increase the populations of *Lactobacillus* and *Bifidobacterium*, reduce the abundance of pathogenic *Colibacillus* in the intestines	*in vivo*	Guo et al. (2022)
UC	*Panax ginseng* C. A. Mey	inhibit inflammation, remodel the intestinal barrier, reduce intestinal epithelial permeability by increasing the abundance of *Akkermansia* and Lachnospiraceae_NK4A136_group while decreasing *Bacteroides* and *Helicobacter*	*in vivo*	Li et al. (2024)
Spleen deficiency syndrome	*Panax ginseng* C.A.Mey	increase the abundance of *Lactobacillus johnsonii* and *Lactobacillus_taiwanensis*	*in vivo*	Dong et al. (2024)
Chronic gastritis	*Codonopsis radix*	suppress the local gastric inflammation and inflammations in peripheral blood leukocytes	*in silico*	Wang et al. (2020)
UC	*Panax ginseng* C.A.Mey	ameliorate inflammation by improving the content of slgA in intestinal mucosa and the IL-2 level in the intestinal tissue	*in vivo*	Yu et al. (2016)
UC	*Panax ginseng* C.A.Mey	exert anti-inflammatory effect by reducing the expression of TNF-α, IL-1β, IL-6, and NO	*in vivo*	Kan et al. (2023)
UC	*Panax ginseng* C.A.Mey	reduce the production of Neutrophil Extracellular Traps through IL1B and TNF	*in vivo*	Zhang et al. (2023)
None	*Panax ginseng* C.A.Mey	improve the TNF-*α* production and NO production of macrophages	*in vitro*	Gao et al. (2019)
Spleen deficiency syndrome	Not mention	improve immune function of the rat through influencing the JAK-STAT signal pathway	*in vivo*	Xiong and Qian (2013)
Intestinal obstruction	*Panax ginseng* C.A.Mey	improve the immunity system by improving the immunoglobulins, complement components, CD4^+^ and CD4^+^/CD8^+^ and reducing the TNF-α and CD8^+^	*in vivo*	Li et al. (2017)
None	*Panax ginseng* C.A.Mey	enhance the phagocytosis and increase the NO production and TNF-α level	*in vitro*	Ji et al. (2017)
Spleen deficiency syndrome	*Panax ginseng* C.A.Mey	increase the expression of T lymphocyte cells and repair the intestinal barrier	*in vivo*	Ma et al. (2021)
Intestinal obstruction	*Panax ginseng* C.A.Mey	regulate the adaptive immune response by reducing the number of CD3^+^T cells and CD8^+^T cells while increasing the number of CD4^+^T cells. promote the recovery of the integrity of the small intestine	*in vivo*	Yu et al. (2014)
Spleen deficiency syndrome	*Panax ginseng* C.A.Mey	repair intestinal epithelium injury through modulation of the FAK/PI3K/Akt signaling pathway	*in vivo* and *in vitro*	Ma et al. (2023)
Wounded intestinal epithelial cells	*Codonopsis radix*	promote intestinal restitution by increasing expression of genes coding for ion channels and transporters	*in vitro*	Liu et al. (2005)
Wounded intestinal epithelial cells	*Panax ginseng* C.A.Mey	promote intestinal epithelial restitution by regulating cellular levels of STIM1 and STIM2	vivo and *in vitro*	Shi et al. (2019)
Intestinal epithelial barrier dysfunction	*Panax ginseng* C.A.Mey and *Codonopsis radix*	attenuates the intestinal barrier dysfunction by inhibiting NF-κB p65-mediated phosphorylation of myosin light chain and myosin light chain kinase	*in vitro*	Lu et al. (2018)

### Regulating the intestinal flora

Microbiota plays a critical role as an important ecosystem in maintaining health by regulating immunity, metabolism, endocrinology, and providing protection against pathogen invasion. However, the intestinal flora is susceptible to dysbiosis caused by various external and internal factors, resulting in gastrointestinal dysfunction. Thus, improving the gut microbiota is essential for understanding the pathogenesis and treatment of gastrointestinal diseases.

Numerous studies have demonstrated that TCM can positively modulate the intestinal environment by influencing gut microbiota metabolites (Yue et al., 2019). To explore the effects of SJZD on gut microbiota, an analysis of ingredients and reaction pathways was conducted to elucidate its impact on gut microorganisms. The findings revealed a significant increase in the abundance of *Bifidobacterium_pseudolongum* with no observed decrease in the abundance of any other species following the administration of SJZD (Zhang Y. et al., 2022). Moreover, SJZD was utilized to investigate its potential anti-diarrheal effects in cases of antibiotic-associated diarrhea. The results revealed that SJZD effectively promoted the growth of beneficial flora by increasing the populations of *Lactobacillus* and *Bifidobacterium*, while simultaneously reducing the abundance of pathogenic *Colibacillus* in the intestines. These actions contributed to alleviating diarrhea symptoms, regulating the gut flora, and maintaining the integrity of intestinal villi (Guo et al., 2022). UC is associated with microbial dysregulation, making the modulation of intestinal flora a critical approach for its treatment. Research has shown that SJZD can reduce inflammation, restore colorectal barrier function, and enhance intestinal permeability in mice with UC by regulating the levels of gut microbiota, specifically *Alistipes*, *Akkermansia*, and Lachnospiraceae_NK4A136_group (Li et al., 2024). The non-polysaccharide components of SJZD, including flavonoids, saponins, and terpenoids, serve as the pharmacodynamic basis for its efficacy. These non-polysaccharides can alleviate gastrointestinal-nervous system dysfunction by modulating the microbiota-gut-metabolites axis, resulting in an increased relative abundance of beneficial probiotics such as *Lactobacillus johnsonii* and *Lactobacillus_taiwanensis* (Dong et al., 2024). These findings suggest that the regulation of intestinal flora is a vital mechanism through which SJZD exerts its therapeutic effects on gastrointestinal diseases.

### Anti-inflammation

Inflammation plays a crucial role in the body’s immune defense, aiding in the eradication of pathogens, tissue repair, and regeneration (Grivennikov et al., 2010). However, chronic inflammation has been associated with the development and progression of several gastrointestinal disorders, including reflux esophagitis, gastritis, gastrointestinal cancer, inflammatory bowel disease, and diarrhea, among others. Consequently, anti-inflammatory interventions are essential in the treatment of these conditions.

Persistent inflammation of the gastric mucosa is recognized as a crucial contributor to the development and progression of gastritis. Research has demonstrated that SJZD effectively alleviates local gastric inflammation and reduces inflammation in peripheral blood leukocytes, thereby providing relief for chronic gastritis (Wang et al., 2020). Inflammation plays a pivotal role in the development of UC, which is characterized as a form of localized recurrence of intestinal inflammatory disease. A study has revealed that SJZD effectively enhances the inflammatory response induced by DSS in rats with UC. This beneficial effect is achieved through improvements in secretory IgA levels and the IL-2 level in the intestinal tissue (Yu et al., 2016). Subsequent investigations have provided additional evidence that SJZD effectively reduces the expression of pro-inflammatory cytokines such as TNF-α, IL-1β, IL-6, and NO, thereby exerting its anti-inflammatory effect and alleviating ulcerative colitis. Among its components, R1, Rg2, and Rb3 demonstrate the most pronounced impact on the anti-inflammatory action (Kan et al., 2023). Neutrophil extracellular traps contain various inflammatory factors and proteins that play a crucial role in the intestinal immune imbalance associated with UC. These factors act as triggers for inflammatory signaling pathways, contributing to intestinal mucosal inflammation in UC (Drury et al., 2021). SJZD treats UC by reducing the levels of intestinal neutrophil extracellular traps, primarily targeting IL1B and TNF (Zhang et al., 2023). In conclusion, SJZD exhibits promising anti-inflammatory activity, making it a potential candidate for future exploration and discovery of novel anti-inflammatory agents.

### Regulating the immune response

Gastrointestinal immune homeostasis plays a vital role in human physiology, as it maintains a delicate equilibrium between immune responses against invading pathogens and tolerance towards symbiotic bacteria. Intestinal diseases, including inflammatory bowel disease (Hu et al., 2023), gastrointestinal neoplasms (Mima et al., 2021), and gastritis (Yang and Hu, 2022), are closely associated with immune dysfunction. This association leads to various alterations in immune responses, impaired barrier function, and abnormal activation of immune cells within the gastrointestinal tract.

Spleen deficiency is frequently associated with immune dysfunction. Research indicates that one of the mechanisms by which SJZD exerts its spleen-tonifying and Qi-replenishing effects is through the modulation of immune function, specifically by enhancing the production of TNF-α and NO in macrophages (Gao et al., 2019). To investigate the role of SJZD in immune regulation, rats were administered SJZD. The results elucidated that SJZD enhances rat immune function by modulating the genetic expression of the JAK-STAT signaling pathway (Xiong and Qian, 2013). In other studies, SJZD was applied for treatment of post-operative ileus. Results demonstrated that SJZD has a moderating effect on immune function by improving the immunoglobulins, complement components, CD4^+^ and CD4^+^/CD8^+^, and reducing the TNF-α and CD8^+^ (Li et al., 2017). Furthermore, several studies investigated the immunomodulatory effects of polysaccharides present in SJZD. The findings revealed that these polysaccharides significantly enhanced macrophage phagocytic activity, stimulated the production of NO, and elevated the level of TNF-α (Ji et al., 2017). Similarly, another study demonstrated that a specific active polysaccharide known as S-3, found in SJZD, augmented intestinal immunity by upregulating the expression of T lymphocyte cells (Ma et al., 2021). These findings indicate that SJZD can target the immune system among its multiple targets.

### Promoting intestinal mucosal restitution

The mucosal barrier assumes a pivotal role as the gastrointestinal tract’s primary defense mechanism, safeguarding against an array of dysfunctions (Sánchez de Medina et al., 2014). Impairment to this barrier constitutes a noteworthy driver of gastrointestinal disturbances, encompassing infections, gastrointestinal inflammatory diseases, and tumors (Dahiya and Nigam, 2023; Song et al., 2023). It is essential to preserve the integrity of the mucosal barrier to ensure optimal gastrointestinal function and impede the onset or progression of these disorders.

In a study examining the impact of SJZD on the restoration of intestinal function in a rabbit model following obstruction relief, the application of SJZD yielded significant outcomes. The findings demonstrated that SJZD effectively facilitated the restoration of intestinal function through multiple mechanisms, including reducing intestinal mucosal permeability, enhancing the secretion of intestinal mucins, and promoting the recovery of small intestine integrity (Yu et al., 2014). Furthermore, network pharmacology and experimental studies have demonstrated that the active components of SJZD possess the ability to repair intestinal epithelium injury induced by spleen deficiency syndrome through modulation of the FAK/PI3K/Akt signaling pathway (Ma et al., 2023). Polysaccharides are abundantly present in Chinese herbs and play a significant role in various physiological functions. As the principal component of SJZD, studies have revealed that these polysaccharides can promote intestinal restitution and provide protection against indomethacin-induced damage to intestinal epithelial cells by enhancing the expression of genes coding for ion channels and transporters (Liu et al., 2005). Intestinal epithelial cell migration is a crucial mechanism in the healing process of mucosal wounds. Experimental studies have demonstrated that the polysaccharides present in SJZD exert a positive effect on intestinal epithelial restitution by differentially regulating the cellular levels of STIM1 and STIM2. Specifically, these polysaccharides stimulate the translocation of STIM1, facilitate the association between STIM1 and TRPC1, and decrease the levels of both STIM1 and STIM2 (Shi et al., 2019). Additionally, another study has demonstrated that the polysaccharides in SJZD can alleviate the impairment of the intestinal epithelial cell barrier function induced by TNF-α by inhibiting NF-κB p65-mediated phosphorylation of myosin light chain and myosin light chain kinase (Lu et al., 2018). In summary, SJZD enhances intestinal mucosal restitution through various mechanisms, highlighting its therapeutic potential in gastrointestinal disorders.

## Conclusion

Digestive diseases encompass a variety of conditions that have the potential to disrupt the normal digestive process, resulting in a spectrum of health effects that can range from mild to severe. It is crucial to emphasize the significance of early diagnosis, appropriate management, and regular monitoring in order to maintain optimal digestive health. Evidence has shown that TCM provides effective therapeutic outcomes for digestive diseases, with its multi-component, multi-target, and multi-pathway approach to overall regulation. As a classical prescription in TCM, SJZD has achieved good curative effects in gastrointestinal disorders in clinical practice due to its multicomponent and multitarget characteristics. In this review, we provide a comprehensive summary of the clinical research advancements surrounding the utilization of SJZD in the treatment of prevalent digestive system diseases. The therapeutic mechanism of SJZD in addressing these disorders is also analyzed, highlighting its significant role in regulating intestinal flora, mitigating inflammation, modulating the immune response, and facilitating mucosal repair.

Although significant progress has been made in exploring the mechanisms by which SJZD prevents and treats digestive diseases, several shortcomings persist. There are only a limited number of prospective, multi-center, and large-sample controlled studies available. Furthermore, as a compound prescription, current research on SJZD primarily examines its mechanisms of action from the perspective of the entire formula or analyzes the effects of individual Chinese herbs and herbal monomers. However, there is a notable lack of experimental studies investigating the interactions among the various components. Given the importance of SJZD in managing gastrointestinal disorders, further investigation into its clinical effects and underlying mechanisms is crucial. Future research should prioritize the clinical translation of SJZD, providing new insights and references for its application in the prevention and treatment of gastrointestinal inflammatory diseases, functional disorders, and tumors. Such efforts will enhance our understanding and optimize the therapeutic use of SJZD in gastrointestinal diseases.

## Abbreviations

SJZD, Sijunzi Decoction; TCM, Traditional Chinese medicine; FD, Functional dyspepsia; IBS, Irritable bowel syndrome; UC, Ulcerative colitis.
